# Brief intervention to reduce risky drinking in pregnancy: study protocol for a randomized controlled trial

**DOI:** 10.1186/1745-6215-13-174

**Published:** 2012-09-24

**Authors:** Graeme B Wilson, Ruth McGovern, Grace Antony, Paul Cassidy, Mark Deverill, Erin Graybill, Eilish Gilvarry, Moira Hodgson, Eileen FS Kaner, Kirsty Laing, Elaine McColl, Dorothy Newbury-Birch, Judith Rankin

**Affiliations:** 1 Institute of Health & Society, Baddiley-Clark Building, Newcastle University, Richardson Road, Newcastle upon Tyne, NE2 4AX, UK; 2 Northumberland, Tyne & Wear NHS Foundation Trust, Newcastle & North Tyneside Addictions Service, Plummer Court, Carliol Place, Newcastle upon Tyne, NE1 6UR, UK; 3 Teams Medical Practice, Watson Street, Gateshead, NE8 2PQ, UK; 4 Women’s Services Directorate, Newcastle upon Tyne Hospitals NHS Foundation Trust, Leazes Wing, Royal Victoria Infirmary, Queen Victoria Road, Newcastle upon Tyne, NE1 4LP, UK

**Keywords:** Pregnancy, Alcohol, Screening, Brief intervention, Trial, Midwife, Motivational interviewing, Public health

## Abstract

**Background:**

Risky drinking in pregnancy by UK women is likely to result in many alcohol-exposed pregnancies. Studies from the USA suggest that brief intervention has promise for alcohol risk reduction in antenatal care. However, further research is needed to establish whether this evidence from the USA is applicable to the UK. This pilot study aims to investigate whether pregnant women can be recruited and retained in a randomized controlled trial of brief intervention aimed at reducing risky drinking in women receiving antenatal care.

**Methods:**

The trial will rehearse the parallel-group, non-blinded design and procedures of a subsequent definitive trial. Over 8 months, women aged 18 years and over (target number 2,742) attending their booking appointment with a community midwife (n = 31) in north-east England will be screened for alcohol consumption using the consumption questions of the Alcohol Use Disorders Identification Test (AUDIT-C). Those screening positive, without a history of substance use or alcohol dependence, with no pregnancy complication, and able to give informed consent, will be invited to participate in the trial (target number 120). Midwives will be randomized in a 1:1 ratio to deliver either treatment as usual (control) or structured brief advice and referral for a 20-minute motivational interviewing session with an alcohol health worker (intervention). As well as demographic and health information, baseline measures will include two 7-day time line follow-back questionnaires and the EuroQoL EQ-5D-3 L questionnaire. Measures will be repeated in telephone follow-ups in the third trimester and at 6 months post-partum, when a questionnaire on use of National Health Service and social care resources will also be completed. Information on pregnancy outcomes and stillbirths will be accessed from central health service records before the follow-ups. Primary outcomes will be rates of eligibility, recruitment, intervention delivery, and retention in the study population, to inform power calculations for a definitive trial. The health-economics component will establish how cost-effectiveness will be assessed, and examine which data on health service resource use should be collected in a main trial. Participants’ views on instruments and procedures will be sought to confirm their acceptability.

**Discussion:**

The study will produce a full trial protocol with robust sample-size calculations to extend evidence on effectiveness of screening and brief intervention.

**Trial Registration:**

Current Controlled Trials ISRCTN43218782

## Background

This project focuses on the reduction of risky alcohol consumption during pregnancy. Risky drinking refers to a level of consumption that increases the likelihood of health problems (hazardous drinking), or that has already caused such problems (harmful drinking), and a pattern of high-intensity drinking associated with intoxication or drunkenness (binge drinking) [1]. Around a third of women drink more than the medically recommended levels [2]. Although older and more affluent women tend to drink more frequently than younger and less affluent women, the latter tend to binge drink (around a fifth of women aged 16 to 44 years binge drink [2]). Binge drinking is strongly associated with sexual risk-taking and unintended pregnancy [3]. Around a third of pregnancies are reported as unintended, but this can be as high as 70% for single women [4] and 82% for those living in impoverished, urban communities [5]. Thus a large number of pregnancies, particularly those in women from lower socio-economic positions, are likely to be significantly related to alcohol [6].

Although most women report that they abstained or reduced alcohol consumption during pregnancy, 8% of recently pregnant women report exceeding the medically recommended guidelines for low-risk drinking in pregnancy [7]. Of the estimated 723,000 births registered in England and Wales in 2010 [8], about 58,000 may have been significantly exposed to alcohol. The teratogenic effects of heavy alcohol consumption are well known [9]. Experts agree that there is a dose-dependent effect of alcohol on fetal and child development [10]. In addition, the timing of alcohol exposure is crucial [9]. Both the first and third trimester of pregnancy in humans are vulnerable periods for central nervous system development [9,11,12]. Thus, although intervention before or very early in pregnancy gives the best opportunity of reducing harm to the developing fetus, reducing alcohol consumption at any stage in pregnancy will have beneficial effects. The likely under-reporting of fetal alcohol effects in the admissions data of UK hospitals makes it unclear whether these data are similar to the higher rates in deprived communities reported from the USA [13-15].

Although recent UK government strategy aims to raise awareness of health risks around alcohol use during pregnancy [16], there is currently no formal program of screening or alcohol intervention during antenatal care in England. Although most women recall alcohol being mentioned by midwives, this seems to have little effect on their drinking behavior, in part because women perceive that they often receive conflicting or unclear messages from health professionals [7]. There is therefore a need for clear, consistent, and effective alcohol advice in antenatal care. A large and robust evidence base supports screening and brief alcohol intervention in other populations. In this secondary preventive strategy, based on social cognitive and social learning theories, risky drinkers receive short advice or counseling, focusing on both personal and contextual factors to promote reduced drinking [17,18]. A recent Cochrane Collaboration meta-analysis identified 29 randomized controlled trials (RCTs) in primary care, and found consistent positive effects of brief interventions compared with control conditions in terms of weekly reductions in alcohol consumption [19]. However, the authors noted that there was a lack of data relating specifically to women. A more recent Cochrane Collaboration review of pre-pregnancy health-promotion interventions identified just one alcohol-specific trial [20], which was based in the USA [21]. Although positive effects were reported for brief intervention, these disappeared in the sensitivity analysis [21]. Thus, brief intervention research focused on antenatal care is urgently needed.

Five brief intervention trials have been conducted with pregnant women in the USA [22-26]. Two trials were small (pilot) studies [23,25], with assessment and/or brief intervention procedures that were much longer in duration (1 to 2 hours) than the time available in antenatal care in the UK [23-25]. Four trials were based in obstetric settings [22-25], and three used specialist practitioners to deliver brief intervention [22,24,25]. Two trials included a single session of brief intervention, one of 10 to 15 minutes [26] and the other of 25 minutes in duration [23]. The former reported significant effects of brief intervention [26], whereas the latter reported reduced drinking in both the control and intervention groups [23]. Finally, one study found that women in antenatal care receiving brief interventions were significantly less likely than women receiving no treatment to have an infant with low birth weight or to suffer preterm labor problems, while another study found positive effects of brief intervention on prenatal drinking in women with higher initial rates of consumption [22,23]. Most recently, a trial of brief interventions in antenatal clinics in South Africa found that women who received a brief intervention had significantly greater reductions in scores on the Alcohol Use Disorders Identification Test (AUDIT) screening tool [27] by their third trimester, compared with women receiving assessment only [28]. Taken together, this evidence suggests that brief intervention is a promising approach for alcohol risk reduction in antenatal care. However, it is necessary to establish if evidence from the USA and elsewhere is applicable to a UK context with marked cultural and health-system differences.

New guidance from the Medical Research Council on developing and evaluating complex interventions [29] is intended to help institute appropriate methods in such research and to enhance the usefulness of its evidence. The guidance points out the potential for evaluations of complex interventions such as brief interventions to be undermined by problems that could be highlighted in a pilot study. Piloting is therefore recommended, but should not be used to test a hypothesis [30]. The pilot study should establish the acceptability of procedures, identify likely recruitment and retention rates, and inform a sample-size calculation for a definitive trial [29]. Because brief interventions to reduce alcohol consumption have not been tested previously in UK antenatal care, a pilot study for such research is proposed on this basis.

### Aim

This pilot study aims to investigate whether it is possible to recruit and retain pregnant women in an RCT of brief intervention aimed at reducing risky drinking in women receiving antenatal care.

The information gathered from this intervention platform research will inform the development and conduct of a future definitive trial.

The specific objectives of this pilot trial are: 1) To conduct an external (rehearsal) pilot RCT comparing brief intervention with standard advice about alcohol in antenatal care; 2) to estimate patient eligibility, recruitment, randomization, retention, and response rates to inform a future definitive trial; 3) to develop methods and instruments for data collection for an economic evaluation of brief intervention in a definitive trial; and 4) to develop the protocol for a definitive trial evaluating the effect of brief alcohol intervention compared with standard advice in antenatal care.

## Methods/Design

### Setting

The trial setting is that of community antenatal care. Under National Health Service (NHS) antenatal care in the UK [31], women initially receive advice from a general practitioner (GP) or midwife about lifestyle, folic-acid supplements, food hygiene, and antenatal screening. A booking appointment subsequently takes place during the first trimester at the woman’s home or in a clinic at the GP surgery. At this meeting, the community midwife provides more detailed information on fetal development, procedures, and resources; carries out assessments to develop an individual care plan; and offers lifestyle advice, screening tests, and an ultrasound scan (carried out at 18 to 20 weeks if desired). Appointments then take place every 2 to 3 weeks from week 25 of pregnancy, in which the midwife carries out routine tests and offers staged advice as appropriate.

For this study, midwives (n = 31) working from four bases of a community midwifery service are recruiting patients over an 8-month period from January 2012. Between them, these midwives provide antenatal care for 36 general practices in Newcastle upon Tyne in north-east England (population 2,607,000; annual live births 30,826), a geographical area with high rates of alcohol misuse and a culture of heavy drinking [32-34]. The practices comprise 41 branch surgeries with diverse patient profiles (total list size 282,034). Each practice is served by one to three midwives and midwives may routinely provide care to patients at one to three practices, depending on practice list sizes and whether or not the midwife works full-time.

### Design

The pilot trial will rehearse the design and procedures we currently envisage for a subsequent definitive trial, and will thereby test their feasibility and acceptability. It is a parallel-group, non-blinded trial, with midwife as the unit of randomization, comparing 5 minutes of structured advice from a community midwife plus a 20 minute brief intervention delivered by a trained alcohol counselor (intervention group) with the standard advice on drinking in pregnancy delivered by community midwives (treatment as usual). The midwives take consent, and deliver the screening and advice to patients (usual standard care if allocated to control condition, structured advice if allocated to the intervention condition), while a trained alcohol counselor attached to the research team delivers the 20 minute intervention (structured alcohol advice and counseling) to patients in the intervention group only. The brief alcohol intervention utilizes motivational interviewing techniques and a directive client-centered approach, and focuses on both personal and contextual factors in promoting behavior change. Our hypothesis for the definitive RCT will be that brief intervention is more effective and cost-effective at reducing risky drinking in pregnant women than a control condition of usual advice in antenatal care.

### Participants

Participants in this trial are women undergoing routine antenatal care who screen positive for risky alcohol use.

#### Inclusion criteria

Any woman aged 18 years or above attending for her routine antenatal care appointment before 16 weeks of gestation who can and does provide verbal agreement to be screened for alcohol, who screens positive for risky alcohol use during pregnancy (score of three or higher on the consumption domain of AUDIT (AUDIT-C)) [35], and who can and does give written consent to participate in the research will be eligible for inclusion.

#### Exclusion criteria

Women are not eligible if they have pregnancy complications or have a multiple pregnancy; if they do not speak sufficient English to participate, or lack the cognitive capacity to understand the research and what is involved; if they have a history of substance use and/or alcohol dependence; or if they are experiencing a severe mental or physical illness likely to have an effect upon the intervention or on their ability to be followed up. Finally, women who are already participating in other alcohol-related research are excluded from the study.

### Randomization

In anticipation of the main trial employing a cluster randomized design to avoid contamination, the unit of randomization is the midwife. It is not practical for individual midwives to deliver both control and intervention conditions in this trial, as training to deliver structured advice will compromise their ability to deliver treatment as usual. Because community midwives may serve multiple GP practices, each midwife consistently delivers either intervention or treatment as usual.

Before screening of women for the trial, midwives were randomized in a 1:1 ratio to either control or intervention group with no concealment of allocation. They were notified of their allocation and trained in the appropriate procedures.

### Screening

Participating midwives inform all women attending their booking appointment about the research and complete the AUDIT-C screening tool [35] with those women who give verbal consent. In a recent systematic review, AUDIT-C (comprising the first three questions of the full AUDIT tool) was shown to have the best sensitivity and specificity of seven screening instruments in identifying problem drinking during pregnancy [36]. An anonymized eligibility screening form is completed by the midwife for all women to determine whether they should be excluded from the study, in order to ascertain any eligibility biases. A screening log is kept to document non-identifiable characteristics of women invited to participate in the study, in order to ascertain whether there is any participation bias. The research team will support midwives in implementing screening procedures through weekly contact with each midwife base.

### Consent

Community midwives will provide a study participant information sheet to all pregnant women at their booking appointment and explain the document. The patient is allowed to keep this document. Owing to the small participant population, the information sheet and consent form for the study are available in English only. Discussions to allow informed consent take place between the midwives and the potential participants, with opportunity for the participants to ask any questions. Because women will usually be asked about their alcohol consumption in the course of routine antenatal appointments, only verbal consent is sought for screening.

Women eligible to participate in the trial are given a reasonable time within the appointment to decide whether or not they wish to do so. Those wishing to take part are asked to provide written informed consent by initialing, signing, and dating a study consent form, which is witnessed and dated by a midwife with documented, delegated responsibility to do so. Written informed consent is always obtained before any study-specific procedures including collection of baseline data. The right to refuse to participate without giving reasons is respected. The original signed consent form is retained in the investigator site file, with a copy retained in the clinical notes and another copy provided to the participant. The participant is specifically asked to consent to their GP being informed of their participation in the study, so that the GP is aware of the research if the woman discusses it during a GP consultation.

### Interventions

#### Control condition: treatment as usual

Women in the control condition will receive usual care delivered by the midwife. This involves the midwife verbally advising that abstinence from alcohol during pregnancy is recommended, and supplying a copy of the Department of Health booklet *The Pregnancy Book*[37], an advisory publication that is issued to all women in the UK attending booking appointments and that contains advice about alcohol use during pregnancy (on page 32). If the midwife judges the reported rate of binge drinking or current alcohol consumption to be of concern, a referral is made to a drug and alcohol midwife for further assessment, advice, or treatment.

Treatment as usual is delivered at the booking appointment immediately after screening, obtaining written consent, and completing the research questionnaire.

#### Brief intervention condition

Women in the intervention condition receive 5 minutes of simple structured alcohol advice delivered by the midwife, using a purposely designed brief advice tool (see Additional file 1). They retain the advice tool and receive a copy of *The Pregnancy Book*. If the midwife judges the reported rate of binge drinking or current alcohol consumption to be of concern, a referral is made to a drug and alcohol midwife for further assessment, advice, or treatment. These procedures take place at the booking appointment immediately after screening, obtaining written consent and completing the research questionnaire.

Women are then advised by the midwife that a member of the research team will contact them shortly by telephone to offer an appointment for brief alcohol intervention (manual available on request) with a trained alcohol health worker. This intervention takes 20 minutes, and is offered within 2 weeks of the initial consultation at the woman’s GP surgery housing the antenatal clinic.

### Training

Intervention midwives have been trained by the alcohol counselor in the delivery of brief advice and provided with a copy of the intervention manual; control midwives will receive this training once follow-up interviews are completed. The alcohol health worker has regular contact with the intervention midwives to encourage fidelity. The alcohol health worker has been fully trained in delivery of the twenty minute intervention. This training included assessment of competence using the Behavior Change Counseling Index [38]. In addition, the alcohol counselor will receive clinical supervision from the consultant psychiatrist (EGilvarry) and management supervision from the project manager (GW). The alcohol counselor will record all appointment durations along with anonymized procedural notes on a case report form to allow the consistency of length of delivery to be assessed.

### Participant compliance

Where feasible, appointments with the alcohol counselor in practices allocated to the intervention group will coincide with routine clinical appointments within the antenatal clinics, to enhance the likelihood of good compliance. The second consultation is sought within 2 weeks of the initial screening to maximize the likelihood that the participant will return. Non-attendance at an intervention appointment, or non-compliance with an arranged follow-up telephone interview (that is, not answering or not being available at specified time) will prompt up to two follow-up telephone calls from the project team to arrange an alternative appointment within 1 week. Participants’ receipt of brief advice, attendance at intervention appointment, receipt of intervention, and participation in follow-up interviews are recorded on a case report form. Participants will not be offered incentives.

### Measures

#### Baseline

The primary outcomes for this feasibility study are rates of eligibility, recruitment, intervention delivery, and participant retention at follow-up. Eligibility will be established by midwives using a checklist of inclusion and exclusion criteria and the AUDIT-C. Rates of recruitment, intervention delivery, and retention will be determined from case report forms. The alcohol counselor will record whether the alcohol intervention was delivered with the participant.

The trial will also measure the proposed outcomes for a future main trial, allowing us to test the usefulness of measures, obtain an estimate of variability to inform power calculations, and assess the acceptability of trial documents. The primary outcome in a full trial will be the maximum number of standard drinks (units) per drinking occasion during the pregnancy, a continuous measure), with secondary outcomes including alcohol abstinence (binary) in the third trimester and 6 months post-partum. Participants will be asked to complete a questionnaire containing three baseline measures, with their midwife’s assistance if requested.

1 The Timeline Follow-Back (TLFB) questionnaire [39] measures alcohol consumption during pregnancy for two weekly periods. The respondent is asked to complete a simple diary (with visual cues) detailing alcohol use in the preceding week. The preceding week might not be typical (for example, a patient might have reduced their drinking as the booking appointment approached), and there may be a tendency to enhance social desirability in responses. Thus, a second diary is requested covering the heaviest drinking week since becoming pregnant. The TLFB has been used as an outcome measure with pregnant women [23,24,40] previously. Scores from the 7-day version, which we will use to minimize the burden on patients, have been found to be strongly correlated with those gathered within longer timeframes [41-43].

2 The AUDIT-C is completed in respect of the participant’s drinking in the 6 months before pregnancy, because the quantity and pattern of previous drinking is predictive of whether or not pregnant women drink during pregnancy [44]. In a full trial, this information will establish whether effects of the intervention differed according to the level of pre-pregnancy drinking.

3 The EuroQoL EQ-5D-3 L instrument measuring quality of life will be completed as part of the health-economics analysis (see below).

The participant will be given time to complete these questionnaires within the appointment before returning them to the midwife, who will check for completeness and prompt for completion if there are missing items. The midwife will abstract data from the participant’s booking notes on to a case report form, including the participants’ health status (including lifestyle behavior such as smoking and body mass index) and social circumstances (marital and employment status, parity). Non-identifiable residential postcode data will be abstracted from contact details provided by trial participants to derive socio-economic profile via the Index of Multiple Deprivation [45]. This index, developed for the UK government, constitutes a weighted area level aggregation of specific distinct dimensions of deprivation that people may experience.

#### Follow-up

Six months after a baseline interview (during the third trimester), the project manager ascertains whether the participant has experienced a fetal loss or had pregnancy complications diagnosed since her first trimester. In either case, the participant will be excluded from follow-up data collection and will not be contacted. This information will be sought from the Regional Maternity Survey Office (RMSO; http://www.nepho.org.uk/rmso/) and the community midwife in order to minimize the risk of causing distress to the participant by inappropriate telephone contact. The RMSO is a publicly funded organization supported by clinical staff, which collects information from hospitals across the north of England to monitor health outcomes for mothers and babies. The project administrator contacts participants who still meet the criteria for inclusion in their third trimester to arrange a telephone interview with the project manager (GW). In the interview, the project manager will administer the 7-day TLFB questionnaire, the EQ-5D-3 L, and a NHS service use questionnaire. We will also check whether participant follow-up rates vary based on socio-economic profile and drinking status.

At 6 months after the due birth date of the participant, the project manager will again contact the RMSO to check whether the participant has experienced a stillbirth or loss after birth, and any such cases will be excluded from follow-up data collection without further contact. Birth outcome data (covering birth weight, gestational age at delivery, presence of congenital anomaly and fetal loss, or loss after birth if appropriate) will also be abstracted and recorded on a case report form. The project manager will then follow up eligible participants by telephone to ascertain longer-term follow-up rates. The screening and outcome measurement tools (the TLFB, EQ-5D-3 L & NHS service use questionnaires) will be re-administered, and open response questions will be asked about the acceptability of the instruments.

#### Economic evaluation

The pilot study will rehearse how the relative costs and benefits of the intervention compared with the control group will be assessed from the perspective of the NHS in a definitive trial. It will also explore the appropriate use and application of data-collection instruments, to allow the economic analysis for a definitive trial to be structured.

This pilot study will test the collection of health-state utilities for each participant because a cost-utility analysis will be performed in the definitive trial. The main analysis will measure the incremental cost per quality adjusted life year (QALY) at the end of the trial follow-up period. QALYs will be based upon the responses to the EQ-5D-3 L, which is a standardized instrument with a three- level variance for use as a measure of health outcomes. Responses will be gathered at baseline and both follow-ups, and converted into QALYs using the area under the curve method [46].

To obtain the costs of the intervention, participating GP practices will be contacted about the type of room used for the delivery of intervention and any related booking costs. A case report form will be used to record the time for the alcohol counselor to book the intervention and to hold the intervention itself. The costs of intervention-related resource use will be obtained from each participating center or constructed based on the unit costs of health and social care (that is, the personal social services research unit). Within the pilot study, the completeness of data recorded from all sources and the appropriateness of the data-collection tools will be assessed.

Data will be collected at post-partum follow-up interviews regarding the use of NHS resources. Use of primary-care services includes antenatal and postnatal contact with midwives, GPs, practice nurses, community psychiatric nurses, dietitians, social workers and other uses relating to primary care (but not of the use of services specifically for treatment of alcohol misuse). Use of secondary care services includes non-intervention protocol, in-patient visits and stays, out-patient visits, visits to accident and emergency departments, readmissions relating to pregnancy, and other secondary care services (also excluding treatment of alcohol misuse).

### Sample-size considerations

**Figure 1:**
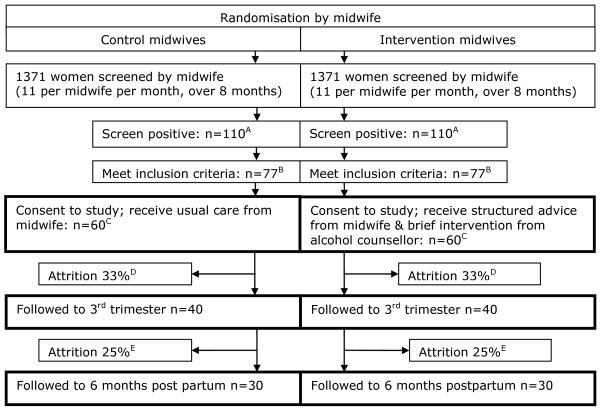
**Flow diagram for the pilot study.** Based on previous data [7], we expect **(A)** 8% to screen positive [7]; **(B)** 10% to be ineligible because of substance use [47] and 20% to be ineligible because of other exclusion criteria. In addition, we expect, based on a comparable US study [26] , **(C)** a 22% refusal rate [27] and **(D)** a 33% attrition rate at first follow-up [46]. At second follow-up we conservatively estimate **(E)** a 25% attrition rate.

This is a pilot trial and therefore a formal power calculation is not required. However, providing data for the power calculation of a definitive trial is an important function of a pilot study, and a minimum number of 30 participants per group is recommended to estimate a parameter for this purpose [47]. We therefore aim to screen 2,742 women to arrive at a sample of 60 providing data at the 6-month follow-up. This is based on an estimate from the UK Infant Feeding Survey 2005 [7] that 220 (8%) of those screened will screen positive. We expect 10% of these to be ineligible because of substance use [48] and 20% because of other exclusion criteria, leaving 70% (154) eligible for the study. We estimate, based on rates in a comparable study in the US [26], that 78% (n = 120) of this remainder will consent to enter the pilot RCT, with 60 participants in each of the intervention and control arms. This should provide data from at least 30 women in each group at follow-up, allowing for 33% attrition in the first wave [47] and 25% in the second wave (Figure 1). In this way, screening and intervention sample sizes will be sufficient to estimate the actual recruitment and retention rates for a UK sample of pregnant women recruited in antenatal care and provide data on the acceptability of study processes and outcome measures [47].

Feasibility criteria are recommended for pilot study design [49]. Support for continuing to a main trial will constitute either: 1) recruitment rate of 78% of those eligible, and retention of 67% and 75% of participants at the two follow-up stages respectively; or 2) a finding that, although not achieved in the pilot phase, these rates would be likely to be achieved with feasible modifications to the trial materials or procedures.

### Planned analysis

As this study is a pilot study, no formal hypothesis will be tested. The aim of the study is to provide robust estimates of the likely rates of recruitment, consent, and retention, and also estimates of the variability of the primary and secondary outcomes to help power calculations for a subsequent full-scale RCT.

The summaries of all baseline characteristics will be presented by treatment group. The summary statistics will be computed for all primary and secondary outcome variables. For all continuous variables, the mean, standard deviation, median, and range will be computed for both the intervention and control groups. For categorical variables, the frequencies and proportions will be calculated. The proportion and the 95% confidence intervals will be presented for the estimates of response, positive screening and eligibility rates, and recruitment and retention rates.

Although the study is not powered, the effect of brief intervention will be analyzed using basic exploratory analysis to compare the intervention and control group using the *t*-test and χ^2^ tests. The outcome analysis will also include the computation of economic analysis in the pilot trial, which will allow the assessment of effectiveness and cost-effectiveness in a full trial. The estimates of the outcome variables of this trial will be used for calculation of the sample size for the full trial. SPSS software (IBM version 17.00; SPSS Inc., Chicago, IL, USA) [50] will be used for all the statistical analyses.

Other aspects of trial design will be confirmed based on the acceptability of the study processes and outcome measures to the pregnant women and the midwives responsible for their care.

### Ethical and research governance approval

The study has been granted a favorable ethical opinion by Newcastle & North Tyneside 2 NHS Research Ethics Committee (MREC reference number: 11/NE/0205).

### Project timescale

The trial duration is 30 months, starting from 1 September 2011. Screening began at the end of January 2012.

## Discussion

Findings from this pilot study will indicate whether and how a definitive trial can establish the effectiveness and cost-effectiveness of screening and brief intervention to reduce heavy drinking among pregnant women in the UK. The outcomes will include the protocol for such a trial, with a sample-size calculation, which can usefully extend the evidence base in this field at an international level.

Screening with brief interventions in healthcare settings is increasingly recognized as an effective approach to improving public health, and is prioritized in the UK government’s recent alcohol strategy [16]. However, knowledge of how effects vary across models and modes of delivery, populations, and settings is limited [51]. Several features of this research are noteworthy in this respect. Alcohol screening and brief intervention involves communicating relatively long-term risk to an individual’s own health, and advising that individual on how to bring their drinking in line with recommended guidelines to reduce that risk [18]. By contrast, brief intervention against heavy drinking in pregnant women seeks to achieve change to a lower level of consumption than the universal guidelines to reduce short-term risk to the pregnancy; even a change during the limited period of pregnancy and early infancy can be considered a successful outcome in this respect. Furthermore, alcohol use is a particularly stigmatized issue to discuss with pregnant women [52-54]. For this reason, compared with the wider population, pregnant women may be more anxious for guidance [55], or more reluctant to engage in talk about alcohol, and these attitudes may affect their willingness to participate in a trial. Finally, much of the evidence base for brief interventions comes from primary healthcare settings. Community midwives have a distinct role and relationship from other health professionals in that they are not treating diseases or disorders, but facilitating the processes of pregnancy and childbirth. However, there are considerable constraints on their time with each patient. Findings from this study will contribute to the broader understanding of screening and brief intervention and related research by indicating how the trial of an intervention focused on pregnancy is likely to be received by women and midwives in the novel healthcare setting of community antenatal care.

### Trial status

The trial is currently recruiting.

## Abbreviations

AUDIT(−C): Alcohol Use Disorders Identification Test (−Consumption); GP: General practitioner; NHS: National Health Service; QALY: Quality Adjusted Life Year; RCT: Randomized controlled trial; RMSO: Regional Maternity Survey Office.

## Competing interests

The authors declare that they have no competing interests.

## Authors' contributions

All authors have made an intellectual contribution to this research trial. As study investigators, EK, JR, EM, KL, MH, and MD, along with collaborators EGilvarry, PC, and DNB, were responsible for identifying the research questions, designing the study, applying for funding and overseeing its implementation. GW is project manager for the study, and RM has responsibility for intervention development and delivery; both have contributed to the development of materials and study implementation. GA has been responsible for randomization procedures and statistical considerations, and EGraybill has contributed to the design of the health-economics component of the study. RM wrote the first draft of the protocol and GW led the drafting of this manuscript, All authors had input into this process and have read and approved the final version of the manuscript.

## Supplementary Material

Additional file 1 RADiANT Brief Advice Tool version 1.0, for use by midwives in the intervention condition. Click here for file
