# Supplementary material for: Brief intervention to reduce risky drinking in pregnancy: study protocol for a randomized controlled trial
Source: Trials. 2012 Sep 24;13:174. doi: 10.1186/1745-6215-13-174 (PMC3543230; doi:10.1186/1745-6215-13-174)
Supplement: Additional file 1 — RADiANT Brief Advice Tool version 1.0, for use by midwives in the intervention condition. [file 1745-6215-13-174-S1.pdf]

Brief Advice RADiANT

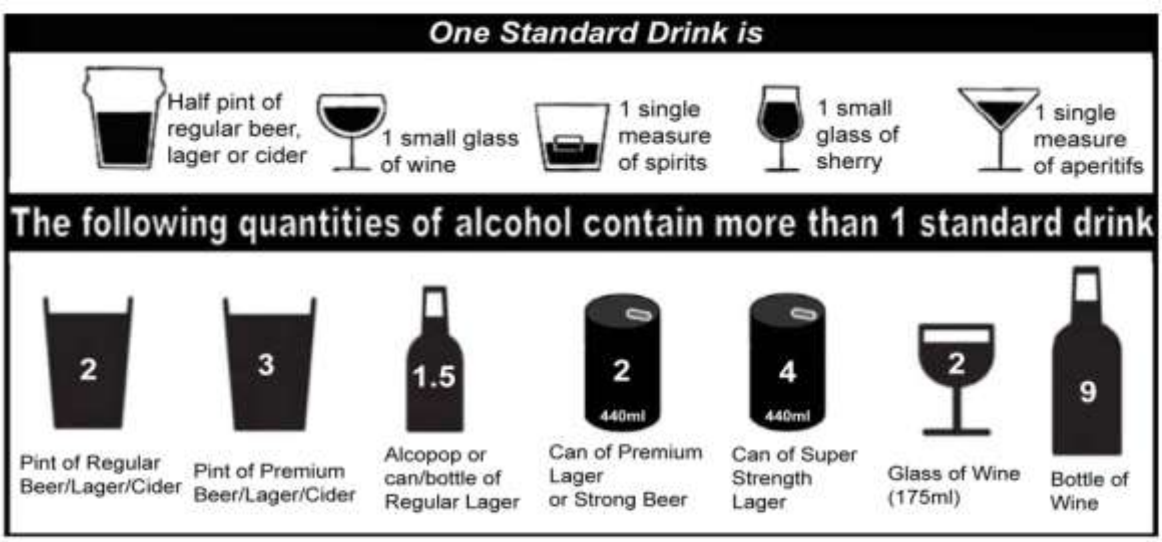

Is your baby at risk from your drinking?

Risk	Women	Possible Risks
LOW RISK	**Avoid alcohol altogether.**<br>If you do choose to drink, you should drink no more than 1-2 units of alcohol once or twice per week.	• Increase likelihood of a healthy pregnancy<br>• Reduce nausea<br>• Reduce risk of harm to your baby<br>• Improve general health and well-being of both you and your baby
**INCREASED/ HIGH RISK DRINKING**	Regularly drinking alcohol<br>Drinking 3+ units in one occasion<br>Drinking on three occasions in one week<br>Getting drunk	**1st trimester – may lead to:**<br>• damage to organs<br>• damage to nervous systems<br>• mental and physical problems<br>• major structural abnormalities<br>• spontaneous miscarriage<br><br>**2nd trimester**<br>• Continued risk of damage to central nervous system<br>• Continued risk of miscarriage<br><br>**3rd trimester**<br>• Disruption to growth & development<br>• Dulled mental abilities<br>• Minor abnormalities<br>• Low birth weight

Alcohol is dangerous to your baby at anytime during your pregnancy and not just in the first 3 months.

Alcohol passes from your blood, through the placenta, to your baby.

A baby's liver is one of the last organs to develop fully and therefore cannot process alcohol as well as you can.

If you drink alcohol during pregnancy your baby could suffer from Fetal Alcohol Sprectrum Disorder (FASD).

What is everyone else like?*

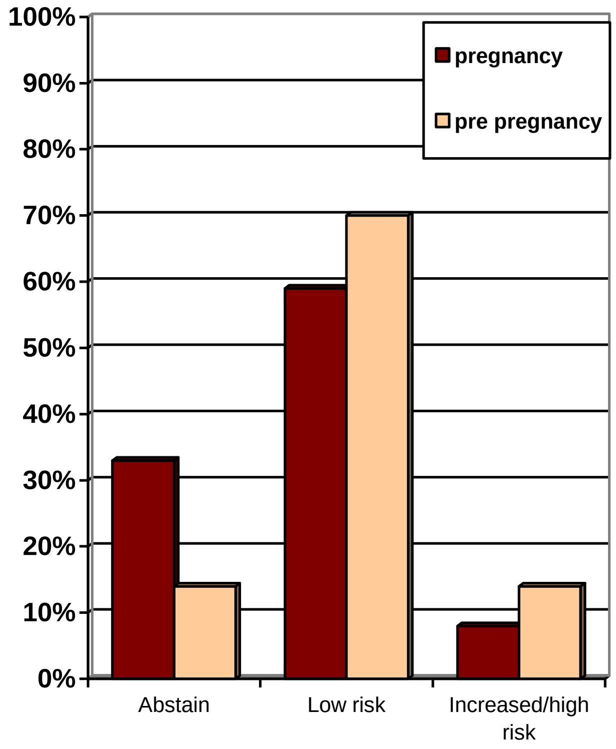

*Source: Bolling, K. et al. (2007) *Infant Feeding Survey 2005*. London: Dept. of Health

What are the benefits of cutting down?

YOU:

Physical

- Sleep better
- More energy
- Better physical shape
- Improved complexion & hair
- Reduced nausea
- Reduce risk of premature birth
- Reduced risk of miscarriage

Psychological/Social/Financial

- Improved mood
- Improved sense of well-being
- Improved relationships
- Save money

Making your plan

- Plan activities and tasks at those times you usually drink
- When bored or stressed take exercise instead of drinking
- Explore other interests such as cinema, exercise, etc.
- Avoid going to the pub after work
- Avoid or limit the time spent with ‘heavy’ drinking friends
- Quench your thirst with non-alcoholic drinks
- If you choose to drink, switch to low alcohol beer/lager/wine
- Take smaller sips
- Avoid drinking in rounds or in large groups
- Don’t allow people to ‘top you up’

YOUR BABY:

Physical

- Reduced risk of damage to central nervous system
- Reduced risk of damage to developing organs
- Reduced risk of structural abnormalities
- Improved growth & development
- Reduced risk of premature birth
- Reduced risk of miscarriage
- reduced risk of FASD

Psychological

- Reduced risk of learning and behavioural disorders

This brief intervention package is based on the Drink-Less programme originally developed at the University of Sydney as part of a W.H.O. collaborative study.

Version 1.0 01/07/11

RADiANT
